# Assessment of the performance of the TGx‐DDI biomarker to detect DNA damage‐inducing agents using quantitative RT‐PCR in TK6 cells

**DOI:** 10.1002/em.22257

**Published:** 2018-11-29

**Authors:** Eunnara Cho, Julie K. Buick, Andrew Williams, Renxiang Chen, Heng‐Hong Li, J. Christopher Corton, Albert J. Fornace, Jiri Aubrecht, Carole L. Yauk

**Affiliations:** ^1^ Environmental Health Science and Research Bureau Health Canada Ottawa Ontario Canada; ^2^ Department of Biology Carleton University Ottawa Ontario Canada; ^3^ Department of Oncology, Lombardi Comprehensive Cancer Center Georgetown University Medical Center Washington District of Columbia; ^4^ Department of Biochemistry and Molecular and Cellular Biology Georgetown University Medical Center Washington District of Columbia; ^5^ Integrated Systems Toxicology Division NHEERL, US‐EPA Durham North Carolina; ^6^ Takeda Pharmaceuticals USA Inc. Cambridge Massachusetts

**Keywords:** toxicogenomics, genotoxicity, transcriptomic biomarker, gene expression signature

## Abstract

Gene expression biomarkers are now available for application in the identification of genotoxic hazards. The TGx‐DDI transcriptomic biomarker can accurately distinguish DNA damage‐inducing (DDI) from non‐DDI exposures based on changes in the expression of 64 biomarker genes. The 64 genes were previously derived from whole transcriptome DNA microarray profiles of 28 reference agents (14 DDI and 14 non‐DDI) after 4 h treatments of TK6 human lymphoblastoid cells. To broaden the applicability of TGx‐DDI, we tested the biomarker using quantitative RT‐PCR (qPCR), which is accessible to most molecular biology laboratories. First, we selectively profiled the expression of the 64 biomarker genes using TaqMan qPCR assays in 96‐well arrays after exposing TK6 cells to the 28 reference agents for 4 h. To evaluate the classification capability of the qPCR profiles, we used the reference qPCR signature to classify 24 external validation chemicals using two different methods—a combination of three statistical analyses and an alternative, the Running Fisher test. The qPCR results for the reference set were comparable to the original microarray biomarker; 27 of the 28 reference agents (96%) were accurately classified. Moreover, the two classification approaches supported the conservation of TGx‐DDI classification capability using qPCR; the combination of the two approaches accurately classified 21 of the 24 external validation chemicals, demonstrating 100% sensitivity, 81% specificity, and 91% balanced accuracy. This study demonstrates that qPCR can be used when applying the TGx‐DDI biomarker and will improve the accessibility of TGx‐DDI for genotoxicity screening. Environ. Mol. Mutagen. 60: 122–133, 2019. © 2018 Her Majesty the Queen in Right of Canada Environmental and Molecular Mutagenesis.

**Abbreviations**
2‐DC: two‐dimensional clustering
2‐DG: 2‐deoxy‐d‐glucose
BSCE: BaseSpace Correlation Engine
CdCl2: cadmium chloride
CPT: campothecin
DDI: DNA damage‐inducing
EMS: ethyl methanesulfonate
ENU: N‐ethyl‐N‐nitrosourea
ETO: etoposide
H_2_O_2_: hydrogen peroxide
K_2_CrO_4_: potassium chromate
MMS: Methyl Methanesulfonate
MOA: mode of action
MTT: 3‐(4,5‐dimethylthiazol‐2‐yl)‐2,5‐diphenyltetrazolium bromide)
MTX: methotrexate
NaAsO_2_: sodium arsenite
NSC: nearest shrunken centroid
PA: NSC probability analysis
PCA: principal component analysis
RT‐qPCR: real‐time quantitative polymerase chain reaction
TGx: toxicogenomics
TSA: trichostatin A

## INTRODUCTION

The application of toxicogenomics (TGx) in chemical toxicity testing has been a continuously developing area of *in vitro* toxicology since the introduction of TGx nearly two decades ago (Nuwaysir et al., 1999; Li et al., 2007; Uehara et al., 2010). TGx biomarkers (i.e., gene expression signatures) are sets of genes that produce characteristic and reproducible transcriptional responses to toxicants with specific modes of action (MOAs) (Lamb et al., 2006; Ellinger‐Ziegelbauer et al., 2009). Thus far, various TGx signatures have been produced that show promise for application in genotoxic hazard identification (Amundson et al., 2005; Ellinger‐Ziegelbauer et al., 2009; Magkoufopoulou et al., 2012; Li et al., 2015). TGx analyses provide transcriptional response information that cannot be obtained using the current standard *in vitro* genotoxicity assays (Thybaud et al., 2007; Zeiger et al., 2015). These biomarkers can be used to measure the initiation of DNA damage response genes and pathways to identify chemicals operating through DNA damage‐inducing (DDI) or potentially other mechanisms. TGx changes may also provide insight into MOA (e.g., identifying chemicals that induce significant amounts of oxidative stress). Thus, TGx can complement standard *in vitro* genotoxicity assays by providing mechanistic context to the observed DNA damage for human‐relevant hazard identification.

In order to address the need for pragmatic TGx tools in genetic toxicology, Li et al. developed the TGx‐DDI transcriptomic biomarker (formerly TGx‐28.65), which classifies chemicals as either DDI or non‐DDI (Li et al., 2015). TGx‐DDI was derived from TK6 human lymphoblastoid cells exposed to 28 representative DDI and non‐DDI agents (the “reference” set) for 4 h. Transcriptional profiles were derived using DNA microarrays, and the Nearest Shrunken Centroid (NSC) method was applied to identify 65 stable and differentially expressed genes that discriminate DDI from non‐DDI exposures. This 65‐gene expression signature (recently modified to 64 genes due to an annotation update) can be used to distinguish DDI from non‐DDI agents based on gene expression. The reference set consists of both DDI and non‐DDI agents with well‐characterized MOAs, which collectively provide a broad representation for each class of chemicals (Li et al., 2015). The DDI group contains agents that induce DNA damage through direct or indirect interactions with DNA, such as DNA alkylators, topoisomerase I/II inhibitors, and DNA antimetabolites. The non‐DDI group includes toxicants that do not interact with DNA, such as endoplasmic reticulum modulators, histone deacetylase inhibitors, and antimitotic agents. DDI classification using the biomarker is made if there is a positive DDI call in at least one of three statistical analyses (referred to as the three‐pronged method)—a probability analysis (PA) based on NSC, principal component analysis (PCA), and 2‐Dimensional Clustering (2‐DC) (Li et al., 2017); otherwise, the agent is classified non‐DDI.

Previous studies have validated the classification capability of the TGx‐DDI biomarker beyond the reference set and TK6 cell line (Buick et al., 2015; Yauk et al., 2016; Buick et al., 2017; Li et al., 2017). The biomarker accurately classifies pro‐genotoxicants in TK6 cells that are metabolically activated with various types and concentrations of rat liver S9, as well as in a metabolically competent human hepatic cell line, HepaRG (Buick et al., 2015; Yauk et al., 2016). In addition, the TGx‐DDI biomarker response is concordant with the micronucleus assay (Yauk et al., 2016), and a case study has shown that the biomarker approach can complement standard methods in regulatory genotoxicity testing (Buick et al., 2017). Recent validation has also demonstrated that the biomarker's classification capability is not limited to DNA microarray technologies, and can be used with new high‐throughput methods to measure gene expression (Li et al., 2017).

Thus far, whole transcriptome analyses using DNA microarrays have facilitated TGx signature development. However, for practical application of the TGx‐DDI biomarker, microarrays lack the accessibility required to accommodate a broad range of users seeking to apply the biomarker in chemical testing. Furthermore, whole transcriptome profiling is unnecessary for TGx‐DDI chemical classification as the biomarker relies on only 64 genes. Quantitative real‐time PCR (qPCR) is a standard method for measuring the expression of select genes, and most molecular biology laboratories are equipped with qPCR instruments. Adapting the biomarker to the qPCR platform will ensure that the TGx‐DDI biomarker approach will be accessible to all molecular biology laboratories.

In the present study, the TGx‐DDI biomarker was measured using TaqMan qPCR gene expression assays in 96‐well plates. The 96‐well qPCR array format provides a customizable platform for analyzing the biomarker genes. We first profiled each agent in the reference set of chemicals (Table 1). In addition, 24 additional chemicals were profiled and classified as external validation of the TGx‐DDI biomarker measured *via* qPCR (Table 2). These 24 chemicals are a subset of the external validation chemicals used in previous validation efforts (Li et al., 2017). Two approaches were applied for chemical classification: the original three‐pronged method (Li et al., 2017), and an alternative method, the Running Fisher test (Kupershmidt et al., 2010). We explored the performance of the biomarker in predicting whether agents are DDI or non‐DDI using qPCR and assessed the use of the alternative classification approach.

**Table 1:** TGx‐DDI Biomarker Reference Agents, Treatment Concentrations, and Vehicle solvents

Class	Mode of action	Chemical names	Concentration	Vehicle solvent
DNA damage‐inducing (DDI)	Alkylating agents	Cisplatin	80 μM	DMSO
		Methyl methanesulfonate (MMS)	40 μg/mL	MeOH
	Topoisomerase I inhibitor	Camptothecin (CPT)	125 nM	DMSO
	Topoisomerase II inhibitor	Etoposide (ETO)	200 nM	DMSO
	RNA/DNA antimetabolites	5‐fluorouracil (5‐FU)	25 μg/mL	DMSO
		Methotrexate (MTX)	100 μM	NaOH
	DNA antimetabolites	Arabinofuranosyl cytidine (AraC)	50 μM	H_2_O
		Hydroxyurea	0.5 mM	H_2_O
	DNA strand break induced by other mechanisms	Gamma irradiation	4 Gy	Media
		Bleomycin	10 μg/mL	H_2_O
		Hydrogen peroxide	80 μM	H_2_O
	Heavy metals	Cadmium chloride	50 μM	H_2_O
		Potassium chromate (IV)	100 μM	H_2_O
		Sodium arsenite	20 μM	H_2_O
Non‐DNA damage‐inducing (non‐DDI)	Antimitotic agents	Colchicine	250 ng/mL	EtOH
		Docetaxel	50 nM	DMSO
		Paclitaxel	50 nM	DMSO
		Vinblastine	200 ng/mL	DMSO
	Histone modification inhibitors	Trichostatin A (TSA)	20 ng/mL	DMSO
		Apicidin	1 μg/mL	DMSO
		HC Toxin	20 ng/mL	MeOH
		Oxamflatin	1 μM	DMSO
	Endoplasmic reticulum modulator	Tunicamycin	2.5 μg/mL	EtOH
		Thapsigargin	250 nM	Acetonitrile
	Glycolysis inhibitor	2‐deoxy‐d‐glucose (2‐DG)	20 μM	H_2_O
	Electron transport chain uncoupler	Antimycin A	100 μM	EtOH
	Other stresses	Heat shock (47 ° C)	47°C for 20 min	Media
		Ethanol	2%, 4%	Media

The 28 reference agents and the concentrations shown above were previously described by Li et al. (2015).

**Table 2:** External Validation Chemicals, Treatment Concentrations, and Vehicle Solvents

Class	Mode of action	Chemical name	Concentration	Vehicle solvent
Class 1 DDI agents that directly interact with DNA	DNA alkylation	Mitomycin C	10 μM	H_2_O
		Chlorambucil	4 μM	DMSO
		Busulfan	20 μM	Acetone
		ENU	500 μM	DMSO
		EMS	2 mM	H_2_O
		Nitrogen mustard	200 nM	H_2_O
	DNA strand breaks	Bleomycin	10 μM	H_2_O
	Mixed, indirect MOA	Hydroquinone	20 μM	H_2_O
Class 4 Non‐DDI, no interaction with DNA	Receptor tyrosine kinase inhibition	Sunitinib malate	20 μM	DMSO
	Antibiotics	Ampicillin	1 mM	H_2_O
		Erythromycin	500 μM	EtOH
	Acetylcholinesterase inhibition	Methyl carbamate	1 mM	H_2_O
	N/A	*N*‐butyl chloride	1 mM	H_2_O
Class 5 Non‐DDI, known irrelevant *in vitro* positive	Protein synthesis inhibition	Cycloheximide	10 μM	H_2_O
	Protein kinase inhibition	Staurosporine	30 nM	DMSO
	Acetylcholinesterase inhibition	Donepezil	1 mM	DMSO
	ETC uncoupling	2,4‐DNP	1 mM	MeOH
	H+/K + ‐ATPase inhibition	Esomeprazole	200 μM	DMSO
		Rabeprazole	0.8 μM	H_2_O
	GABA receptor activation	Phenobarbital	1 mM	H_2_O
	Angiotensin II inhibition	Olmesartan	0.16 μM	NaOH
	Aromatase inhibition	Exemestane	100 μM	MeOH
	Dopamine receptor activation	Rotigotine	100 μM	DMSO
	Glucocorticoid receptor agonist	Dexamethasone	1 mM	MeOH

The 24 chemicals and the concentrations shown above were previously described by Li et al. (2017).

## MATERIALS AND METHODS

### Cell Culture and Treatments

Cell culture and treatments were performed as described in the original TGx‐DDI publication (Li et al., 2015). RNA from the original exposures in Li et al. was no longer available for this experiment. Thus, a new experiment was conducted to re‐derive cells for the reference set. For this, TK6 cells were exposed to each reference agent and its solvent on two separate occasions to produce two biological replicates of each treatment and control.

For external validation, the same RNA samples isolated by Li et al. (2017) were used; a single replicate of 24 treatments and corresponding controls were previously generated using the same exposure methods as the reference set (Li et al., 2017).

The concentration and vehicle solvent for each treatment are listed in Table 1 (Reference set) and Table 2 (External validation set). Overall, the treatment chemicals consisted of the 28 reference agents and 24 of the external agents used in the 2015 and 2017 studies by Li et al. (2015, 2017). Eight DDI agents (called Class 1 chemicals in Li et al., 2017), five non‐DDI agents (Class 4 chemicals in Li et al., 2017), and 11 chemicals that cause chromosomal aberrations *in vitro* but not *in vivo* (Class 5 chemicals from Li et al., 2017) constituted our external validation set.

Briefly, TK6 cells were obtained from American Type Culture Collection (ATCC# CRL‐8015; ATCC, Manassas, VA) and cultured in RPMI 1640 medium with 10% fetal bovine serum. All treatment chemicals were purchased from Sigma‐Aldrich (St. Louis, MO) except for potassium chromate and cadmium chloride, which were purchased from J. T. Baker (Phillipsburg, NJ). The concentrations were the same as the concentrations optimized by Li et al. (2015); the concentrations were selected based on the cell viability measured by the MTT (3‐(4,5‐dimethylthiazol‐2‐yl)‐2,5‐diphenyltetrazolium bromide) assay and the level of stress response measured using the expression of three indicator genes, *ATF3, GADD45A,* and *CDKN1A* (Li et al., 2015). The selected concentrations were those that induced the largest fold changes in the three indicator genes while maintaining the cell viability above 50% at 24 h. Cells were treated with each agent in the exponential growth phase at a density of 4–5 × 10^5^ cells/mL for 4 h at 37°C before subsequent harvest and RNA extraction. The solvent control samples were prepared concurrently with the treated samples by treating the cells with an equal volume of the vehicle solvent as the treatment.

### RNA Isolation and Quality Assessment

To extract and purify total RNA, the RNeasy Mini Kit (Qiagen, Toronto, ON, Canada) was used following the manufacturer's protocol. The quantity and quality of each extracted RNA sample were assessed using a NanoDrop ND‐100 spectrophotometer (Thermo Scientific, Burlington, ON, Canada) and an Agilent 2100 Bioanalyzer (Agilent Technologies, Mississauga, ON, Canada). All RNA samples used had A260/280 absorbance ratios of ≥2.0 and RNA integrity number between 7.5 and 10.

### Complementary DNA Synthesis and TaqMan qPCR Assays

RNA was reverse transcribed to cDNA using the SuperScript IV First‐Strand Synthesis System (Invitrogen, Burlington, ON, Canada). In each 20 μL cDNA synthesis reaction, 500 ng of RNA was input. For each of the 28 reference agents and seven vehicle controls, five cDNA synthesis reactions were performed, using 2.5 μg of RNA in total. The cDNA from the five reactions were pooled to 100 μL and diluted 1:10 in water to a final volume of 1 mL for each 96‐well qPCR array.

The 96‐well qPCR array was constructed from pre‐designed, inventoried TaqMan Gene Expression Assays (FAM‐labeled, MGB probe/primer sets, Applied Biosystems, Burlington, ON, Canada) (list of assays in Supporting Information Table I and plate layout in Supporting Information Fig. 1). Six reference genes were added to the arrays: *HPRT1*, *GUSB*, *GAPDH*, *CASC3*, *18S*, and *EIF2B1*.

Complementary DNA from two biological replicates of the 28 reference samples and the seven vehicle controls, and a single replicate of each of the 24 validation samples and the seven vehicle controls were prepared for qPCR with TaqMan Gene Expression Master Mix (Applied Biosystems) following the manufacturer's instructions for 20 μL reactions. qPCR was performed on the CFX96 Real‐Time System (Bio‐Rad, Mississauga, ON, Canada) under settings for 96‐well plates recommended by Applied Biosystems (Burlington, ON, Canada) (50°C for 2 min, 95°C for 10 min, and 40 cycles of 95°C for 15 s followed by 60°C for 1 min).

### Quantitative RT‐PCR Gene Expression Fold Change Calculation

After performing qPCR for all samples, the biomarker gene set was modified to 61 genes for the analyses, as three genes (*ARRDC4*, *PCDH8*, and *SEMG2*) were excluded from statistical analyses due to Cq (quantification cycle) values above 40 across the majority of samples.

Based on the stability of the Cq value across all samples, the two most stably expressed reference genes, *HPRT1* and *GUSB*, were averaged and used to generate ΔCq values of each reference expression profile. ΔΔCq values were obtained by normalizing each chemical by the corresponding vehicle control. The fold change in each gene was then calculated using the following equation: Fold change = 2^−ΔΔCq^ (Vandesompele et al., 2002). The heatmap of biomarker gene expression under each chemical treatment was generated using the log_2_ fold change. All of the qPCR data are available on NCBI's Gene Expression Omnibus (GEO) under the accession number GSE121532 (URL: https://www.ncbi.nlm.nih.gov/geo/query/acc.cgi?acc=GSE121532).

### Chemical Classification by the Three‐Pronged Approach

The three‐pronged approach involves assessing performance using PCA, 2‐DC, and the NSC PA of the test agent alongside the reference agents (Li et al., 2017).

**Figure 1:**
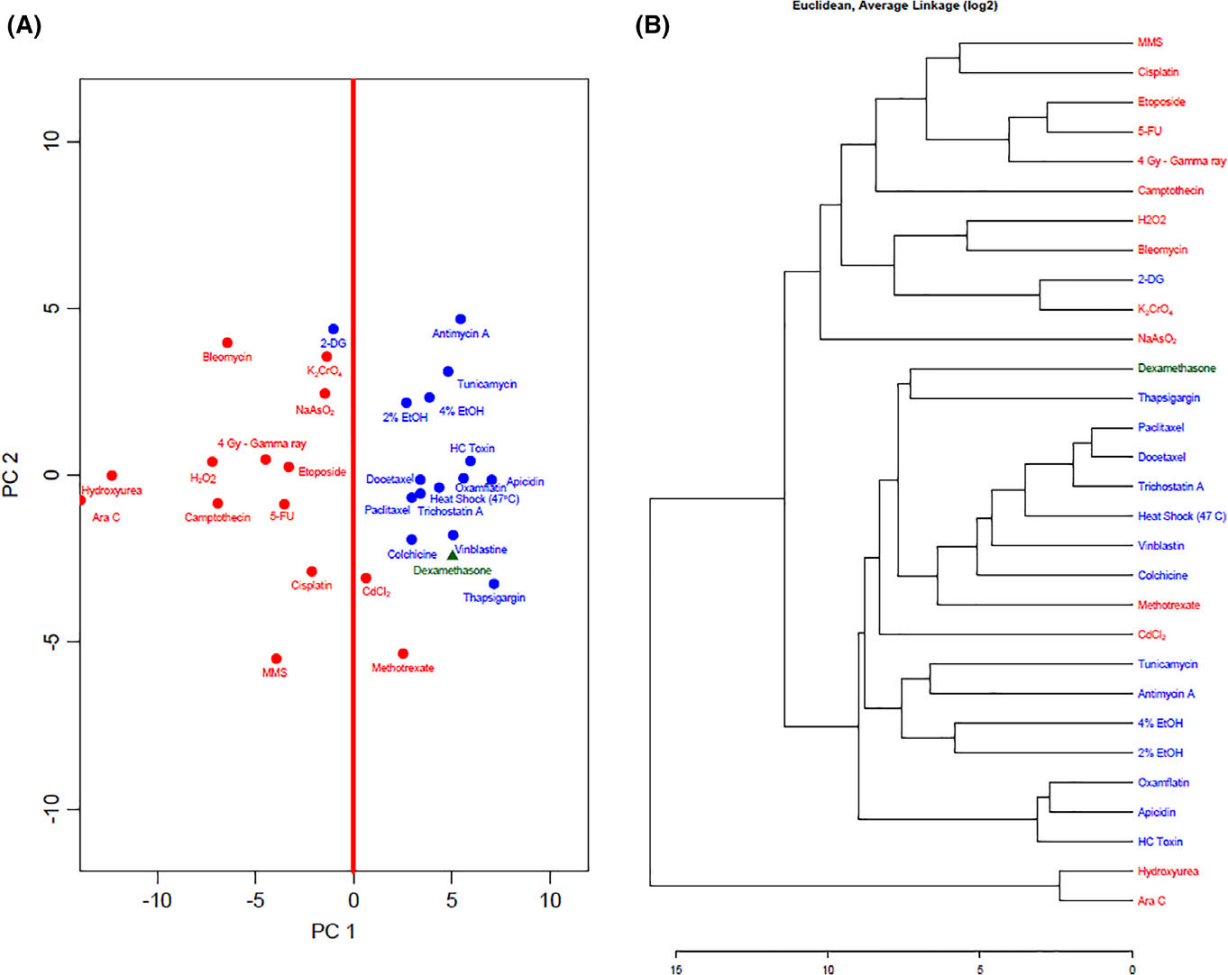
(A) Principal component analysis (PCA) of the qPCR profiles of the reference set and dexamethasone, an external validation agent. (B) Two‐dimensional clustering (2‐DC) of the qPCR profiles of the reference set and dexamethasone. Red font indicates DDI reference agents and blue font indicates non‐DDI agents. Green font represents the external validation chemical.

The PCA was performed using the prcomp in R (www.r-project.org) and a scatterplot was generated to represent the results (Fig. 1A). 2‐DC (Fig. 1B) was performed using the hclust function in R using average linkage and Euclidean distance.

To perform the PA using NSC, the pamr function in the R statistical environment was used (www.bioconductor.org). Briefly, the centroids for each class were generated from the expression profiles of reference chemicals belonging to each class by averaging the expression of each gene across each class (Tibshirani et al., 2002). The PA classifies chemicals based on the similarities in their centroids to that of the reference chemicals. A test chemical was assigned to the class (DDI or non‐DDI) where its probability of membership was >0.90 (Li et al., 2015).

The overall prediction made by the TGx‐DDI biomarker utilized the results from all three analyses. Higher weight was given to results indicating DDI; if one of the analyses indicated that a chemical is DDI, regardless of the other two analyses, the overall prediction was stated as DDI for a conservative assessment of genotoxic potential. Similarly, if one of the analyses indicated non‐DDI and the remaining two were inconclusive, the overall prediction was non‐DDI. Any chemical that lay on the border in the PCA or did not branch with either of the two classes in hierarchical clustering was considered inconclusive (Li et al., 2017).

### Chemical Classification by the Running Fisher Test

The use of the Running Fisher test to determine the predictive accuracy of the original TGx‐DDI biomarker has been described (Corton et al., 2018). Briefly, the TGx‐DDI transcriptomic biomarker was uploaded to the BaseSpace Correlation Engine database (URL: https://www.illumina.com/products/by-type/informatics-products/basespace-correlation-engine.html; formally NextBio) (Kupershmidt et al., 2010) and compared to each list of fold changes derived by qPCR using the Running Fisher test. For each pairwise comparison, the *P*‐value of the Running Fisher test and direction of the correlation were exported. *P*‐values were converted to –Log_10_(*P*‐value)s and those with negative correlations were converted to negative numbers. The reference set of 28 treatments was used to derive a –Log_10_(*P*‐value) cut‐off of 19 that eliminated false negatives. This cut‐off was then applied to the external test set.

### Combining the Classification Approaches in External Validation

Calls made by the individual analyses of the three‐pronged approach and by the Running Fisher test were combined to determine the aggregate analysis that produces the most accurate classifications in external validation for the qPCR platform. The overall predictions made by each combination was determined following the same rule applied in the three‐pronged approach; one DDI call indicated an overall DDI call and if there was no DDI call, the chemical was classified non‐DDI.

## RESULTS

### Classification of  the Reference Set Using the TGx‐DDI Transcriptomic Biomarker

The 28 reference agents and seven vehicle controls were profiled using the 96‐well custom TaqMan TGx‐DDI arrays. Following normalization with the corresponding vehicle controls, the NSC method was applied to generate centroids associated with DDI and non‐DDI classes. The three analyses, namely PCA, 2‐DC, and the PA from the NSC, were then applied to assign each chemical as DDI or non‐DDI.

In the PCA, 2‐deoxyglucose (2‐DG), a non‐DDI agent, clustered with DDI chemicals, and methotrexate (MTX) and cadmium chloride, two DDI agents clustered with non‐DDI chemicals (Fig. 1A); thus, all three were misclassified. The remainder of the reference set clustered within their respective classes. Cadmium chloride, MTX, and 2‐DG were also misclassified in the 2‐DC analysis, where 2‐DG branched with the DDI chemicals while MTX and cadmium chloride branched with the non‐DDI chemicals (Fig. 1B).

**Figure 2:**
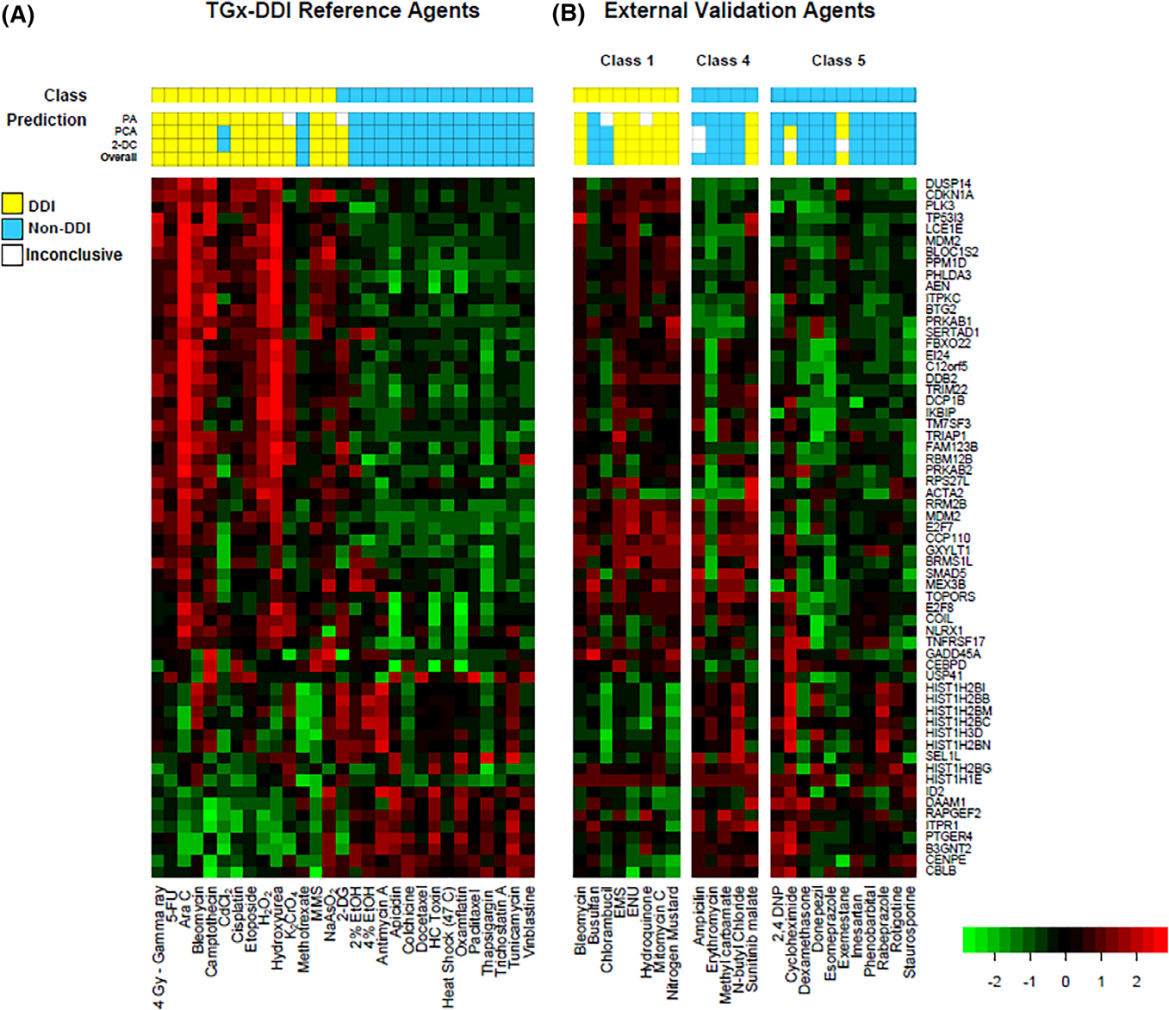
Heatmaps and predictions made by the TGx‐DDI biomarker generated using RT‐qPCR 4 h post‐exposure in TK6 cells. TK6 cells were treated with the 28 reference agents (A) and 24 external validation chemicals (B) listed on the *x*‐axis. The 28‐chemical reference set divides into two classes consisting of 13 DNA damage‐inducing (DDI) and 15 non‐DDI chemicals. The external validation set spans three classes: Class 1 (direct‐acting DDI), Class 4 (non‐DDI), and Class 5 (known irrelevant *in vitro* positive, non‐DDI). The biomarker genes are labeled on the right side of the heatmap. Each square represents a gene's transcript levels relative to controls, with the color of the square indicating up‐regulation (red) or down‐regulation (green) of the gene. The color scale corresponds to the fold change in expression on the log_10_ scale. The “Prediction” bars above each heatmap represent the outcomes of the NSC probability analysis (PA), PCA, and 2‐DC, yellow indicating DDI, blue indicating non‐DDI, and white indicating inconclusive result. The “Class” bar represents the expected classification based on the classifications in Li et al. (2017).

The PA of the reference compound set measured using qPCR (Fig. 2A) produced 26 correct classifications, two inconclusive, and one false negative classification. The two inconclusive results included one DDI chemical, potassium chromate (K_2_CrO_4_), and one non‐DDI agent, 2‐DG. MTX was misclassified as non‐DDI (false negative) in the NSC PA. Cadmium chloride was correctly classified as DDI in the PA; thus, the overall classification of cadmium chloride was DDI, despite the misclassifications in the PCA and the 2‐DC analysis.

Overall, the combined classification approach for the TGx‐DDI biomarker measured using qPCR classified 26 of the 28 agents in the reference set accurately (93% sensitivity; 93% specificity; 93% balanced accuracy; 93% accuracy).

### External Validation of the TGx‐DDI Biomarker Using the Three‐Pronged Approach

For external validation of the TGx‐DDI biomarker measured using qPCR, 24 additional chemicals (Table 2) were profiled and classified using the same methods as the qPCR reference set (Figs. 1 and 2B, and Supporting Information Figure 2).

Of the eight Class 1 agents (i.e., DDI agents) tested, the TGx‐DDI biomarker correctly classified six out of eight external validation chemicals. Although hydroquinone rendered an inconclusive result in the PA, it was correctly classified as DDI in the PCA and 2‐DC analysis (Fig. 2B), leading to a correct DDI classification in the three‐pronged classification approach. Chlorambucil and busulfan were misclassified as non‐DDI, as they were either inconclusive or incorrectly classified as non‐DDI in all three classification methods (Fig. 2B).

Of the five Class 4 agents (i.e., non‐DDI chemicals) tested, one misclassified and one was inconclusive. Sunitinib malate was predicted to be DDI in all three classification methods, resulting in an incorrect overall classification of DDI (Fig. 2B). Although both PCA and 2‐DC analyses were inconclusive for ampicillin, the PA correctly identified it as non‐DDI (Fig. 2B); thus, the overall classification of this chemical was non‐DDI in the three‐pronged classification approach.

Among the 11 Class 5 chemicals (i.e., non‐DDI chemicals that are known to produce irrelevant positive results in *in vitro* genotoxicity tests) tested, two chemicals were misclassified. Exemestane was misclassified as DDI in the PA and PCA, and was inconclusive in the 2‐DC analysis (Fig. 2B). Cycloheximide produced different results in all three classification methods. Although the PA predicted cycloheximide to be non‐DDI and 2‐DC was inconclusive, cycloheximide was close to the cut‐off on the DDI side in the PCA, leading to an overall misclassification as DDI (Fig. 2B).

In summary, classification of chemicals using the TGx‐DDI biomarker measured *via* qPCR accurately classified 18 external validation chemicals—six of the eight Class 1 DDI agents and 13 of the 16 Classes 4 and 5 non‐DDI agents—resulting in a sensitivity of 75%, specificity of 81%, an accuracy of 79%, and a balanced accuracy of 78%.

### Determination of the Predictive Accuracy of the TGx‐DDI Biomarker Using the Running Fisher Test

**Figure 3:**
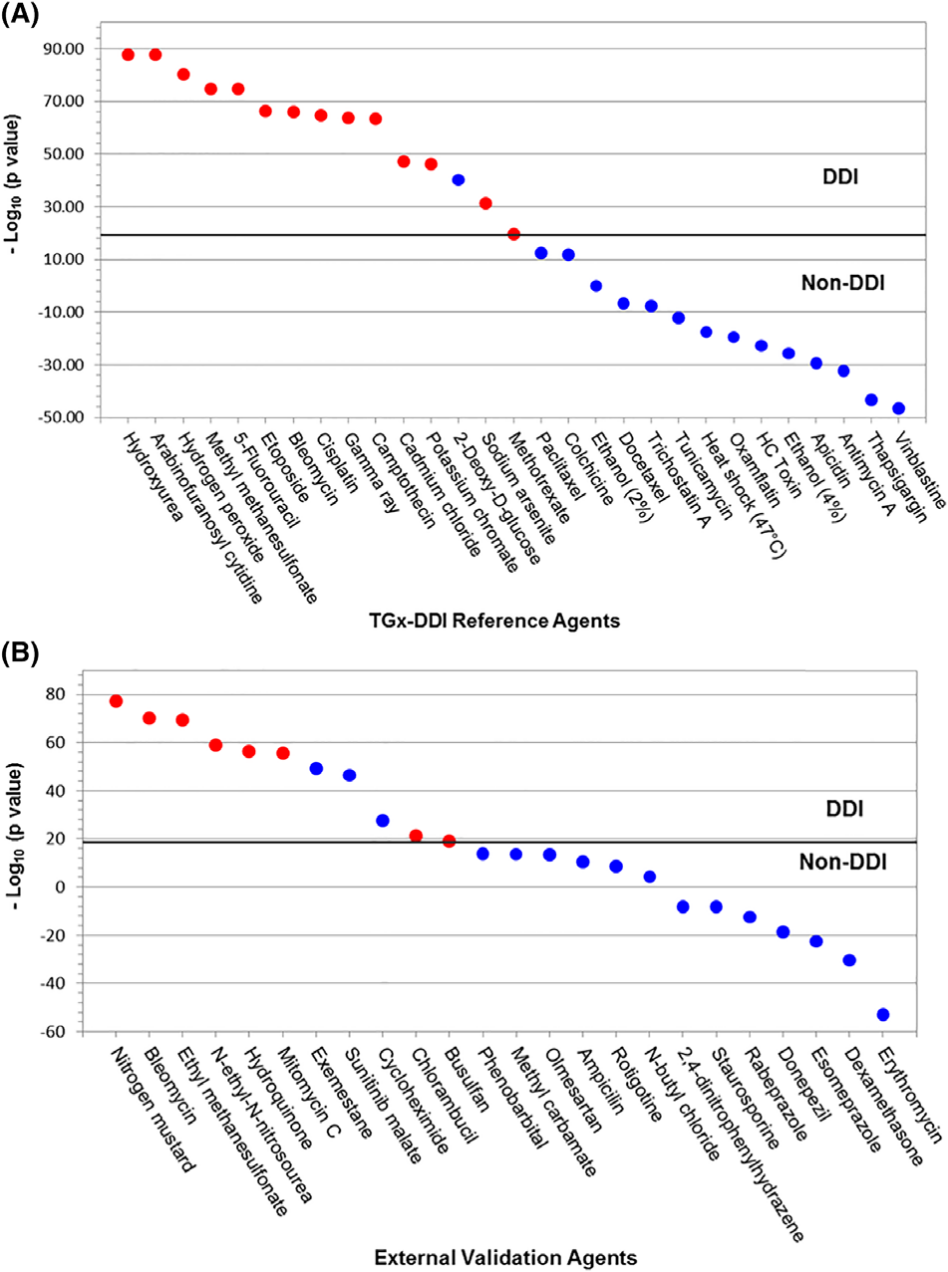
The Running Fisher test results for the 28 reference agents (A) and 24 external validation chemicals (B). Red dots indicate DDI and blue dots indicate non‐DDI agents. Chemicals are ranked by –Log_10_(*P*‐value) from the highest on the left to the lowest on the right. A cut‐off of 19 was derived from the reference set and applied to both datasets to separate DDI and non‐DDI agents (horizontal line). Chemicals above the cut‐off were classified as DDI and ones below were classified as non‐DDI.

The predictive accuracy of the TGx‐DDI biomarker was determined using the Running Fisher test. The reference set of 29 treatments was first used to derive a –Log_10_(*P*‐value) cut‐off that eliminated false negatives (Fig. 3A). Using a cut‐off of 19, there was one false positive, 2‐deoxy‐d‐glucose, in the reference set. Using this threshold, the qPCR TGx‐DDI biomarker demonstrated 100% sensitivity, 93% specificity, and 96% accuracy and balanced accuracy.

The external validation set of 24 treatments was then analyzed. Using the same cut‐off of 19 derived from the reference set, there were three false positives: exemestane, sunitinib malate, and cycloheximide (Fig. 3B). The test thus had a sensitivity of 100% and a specificity of 81%, resulting in an accuracy of 88% and a balanced accuracy of 91% in external validation.

### Combination of Classification Approaches in External Validation

Calls made by the individual analyses of the three‐pronged approach and by the Running Fisher test were combined to determine the best combination of classification methods (Tables 3 and 4). The addition of the Running Fisher test to the three‐pronged approach eliminated two false negative calls for chlorambucil and busulfan, resulting in 100% sensitivity, 81% specificity, 88% accuracy, and 91% balanced accuracy. All combinations containing one of the three analyses of the three‐pronged approach and the Running Fisher test produced the same results as above.

**Table 3:** Calls Made by Different Combinations of Statistical Analyses in External Validation.

	External validation chemicals	Three‐pronged	RF	Three‐pronged + RF	PA + RF	PCA + RF	2‐DC + RF
Group 1 DDI	Bleomycin	DDI	DDI	DDI	DDI	DDI	DDI
	Busulfan	Non‐DDI	DDI	DDI	DDI	DDI	DDI
	Chlorambucil	Non‐DDI	DDI	DDI	DDI	DDI	DDI
	EMS	DDI	DDI	DDI	DDI	DDI	DDI
	ENU	DDI	DDI	DDI	DDI	DDI	DDI
	Hydroquinone	DDI	DDI	DDI	DDI	DDI	DDI
	Mitomycin C	DDI	DDI	DDI	DDI	DDI	DDI
	Nitrogen mustard	DDI	DDI	DDI	DDI	DDI	DDI
Group 2 Non‐DDI (clean genotoxicity profile)	Ampicillin	Non‐DDI	Non‐DDI	Non‐DDI	Non‐DDI	Non‐DDI	Non‐DDI
	Erythromycin	Non‐DDI	Non‐DDI	Non‐DDI	Non‐DDI	Non‐DDI	Non‐DDI
	Methyl carbamate	Non‐DDI	Non‐DDI	Non‐DDI	Non‐DDI	Non‐DDI	Non‐DDI
	*N*‐butyl chloride	Non‐DDI	Non‐DDI	Non‐DDI	Non‐DDI	Non‐DDI	Non‐DDI
	Sunitinib malate	DDI	DDI	DDI	DDI	DDI	DDI
Group 3 Non‐DDI (known *in vitro* positive)	2,4 DNP	Non‐DDI	Non‐DDI	Non‐DDI	Non‐DDI	Non‐DDI	Non‐DDI
	Cycloheximide	DDI	DDI	DDI	DDI	DDI	DDI
	Dexamethasone	Non‐DDI	Non‐DDI	Non‐DDI	Non‐DDI	Non‐DDI	Non‐DDI
	Donepezil	Non‐DDI	Non‐DDI	Non‐DDI	Non‐DDI	Non‐DDI	Non‐DDI
	Esomeprazole	Non‐DDI	Non‐DDI	Non‐DDI	Non‐DDI	Non‐DDI	Non‐DDI
	Exemestane	DDI	DDI	DDI	DDI	DDI	DDI
	Olmesartan	Non‐DDI	Non‐DDI	Non‐DDI	Non‐DDI	Non‐DDI	Non‐DDI
	Phenobarbital	Non‐DDI	Non‐DDI	Non‐DDI	Non‐DDI	Non‐DDI	Non‐DDI
	Rabeprazole	Non‐DDI	Non‐DDI	Non‐DDI	Non‐DDI	Non‐DDI	Non‐DDI
	Rotigotine	Non‐DDI	Non‐DDI	Non‐DDI	Non‐DDI	Non‐DDI	Non‐DDI
	Staurosporine	Non‐DDI	Non‐DDI	Non‐DDI	Non‐DDI	Non‐DDI	Non‐DDI
	Sensitivity (%)	75	100	100	100	100	100
	Specificity (%)	81	81	81	81	81	81
	Accuracy (%)	79	88	88	88	88	88
	Balanced Accuracy (%)	78	91	91	91	91	91

RF = Running Fisher; PA = probability analysis; PCA = principal component analysis; 2‐DC = 2‐dimensional clustering. Chemicals with one or more DDI call was classified DDI, and otherwise, non‐DDI.

**Table 4:** Summary of Sensitivity, Specificity, and Balanced Accuracy of TGx‐DDI Determined by Individual Analyses and Aggregate Analyses in External Validation

	Probability analysis (%)	PCA (%)	2‐DC (%)	Three‐pronged overall (%)	Running Fisher (%)	Three‐pronged + running Fisher (%)
Sensitivity	63	75	75	75	100	100
Specificity	88	75	75	81	81	81
Balance Accuracy	75	75	75	78	91	91

## DISCUSSION

We measured the TGx‐DDI biomarker using TaqMan qPCR assays to test its performance on this widely available gene expression platform. The qPCR expression signature of the 28 reference agents was then used to classify 24 external test agents to test performance. The chemicals were classified using two approaches, the three‐pronged classification strategy (PA, PCA, and 2‐DC) and the Running Fisher test.

Using the three‐pronged classification strategy, the TGx‐DDI biomarker measured using qPCR correctly classified 26 out of the 28 chemicals in the reference set, yielding 93% balanced accuracy; the two misclassifications included one false positive (2‐DG) and one false negative (MTX). The Running Fisher test of the qPCR profiles of the TGx‐DDI reference set demonstrated an improvement in accuracy by classifying 27 reference agents correctly (96%). Although the two classification methods were consistent in the misclassification of 2‐DG, the Running Fisher test produced no false negatives. TGx‐DDI was previously validated on the Nanostring nCounter system (Li et al., 2017), where it fully retained the original classification capability and accurately classified all reference agents using the three‐pronged strategy. Overall, analysis of the reference set of agents suggests that the TGx‐DDI biomarker has comparable performance on microarray, nCounter, and qPCR platforms, with the two former technologies yielding slightly higher accuracies.

The performance of the TGx‐DDI biomarker measured using qPCR was further tested by analyzing an additional 24 chemicals that were previously analyzed by both microarray and nCounter technologies (Li et al., 2017). These agents spanned three classes studied in Li et al.: Class 1 (established DDI agents), Class 4 (established non‐DDI agents that are negative in *in vitro* assays), and Class 5 (non‐DDI agents known to produce irrelevant positive results *in vitro*). Of the 24 chemicals, five were misclassified using the three‐pronged strategy and three were misclassified using the Running Fisher test. The TGx‐DDI biomarker measured using qPCR misclassified two alkylating agents, chlorambucil and busulfan, as non‐DDI in the three‐pronged approach, but both were correctly classified as DDI using the Running Fisher test. The misclassification of non‐DDI chemicals was consistent in both classification approaches; sunitinib malate (Class 4), and exemestane and cycloheximide (Class 5), were classified as DDI. Notably, exemestane, an aromatase inhibitor, was previously misclassified using both microarrays and the nCounter system (there were two and one misclassifications from the microarray and nCounter validation exercises, respectively, for these 24 chemicals). The three misclassified non‐DDI agents from the validation set (sunitinib malate, exemestane, and cycloheximide) and one from the reference set (2‐DG) can alter cell signaling and metabolism; such non‐genotoxic modulation of gene expression may influence the biomarker performance as described previously (Li et al., 2017). Overall, the TGx‐DDI biomarker was able to identify nine out of 11 Class 5 compounds (81%) as non‐DDI, clearly demonstrating its ability to distinguish irrelevant from relevant positives, which highlights the ability of the TGx‐DDI biomarker to inform human relevance as described previously (Li et al., 2017).

Slight discrepancies in biomarker performance across platforms may arise from differences in sensitivity, specificity, and dynamic range of each platform. Compared to both qPCR and nCounter, microarrays have a smaller detection range, and are subject to increased variability in the measurement of weakly expressed genes (Etienne et al., 2004; Richard et al., 2014). In addition, we used manufacturer‐designed TaqMan assays that were designed for maximal coverage of annotated transcripts of each gene and to span exons to prevent contamination of measurements by genomic DNA. Thus, DNA microarray and TaqMan probes may be in different exons of the biomarker genes, which likely introduce variability between the platforms (Etienne et al., 2004). Considering that TGx‐DDI was derived from the NSC identified in the microarray expression profiles, it is not surprising that the expression signature reproduced on qPCR is not identical to the original.

The two different approaches to chemical classification produced similar results. Overall, the Running Fisher test of the TGx‐DDI biomarker measured using qPCR generated no false negatives (100% sensitivity), while the original three‐pronged method produced two false negative calls (75% sensitivity) in external validation, demonstrating increased sensitivity in detecting DDI agents. The Running Fisher test also provided clear calls for chemical classes; whereas, some analyses in the three‐pronged classification strategy produced “unclassified” calls. The Running Fisher test applies a –Log_10_(*P*‐value) cut‐off and classifies chemicals based on their position above or below this threshold, providing dichotomous calls. Overall, the addition of Running Fisher test improved the sensitivity of TGx‐DDI using qPCR. The results indicate that using the TGx‐DDI biomarker in the context of the Running Fisher test might be advantageous for high throughput screening to identify DDI agents through assessment of gene expression by qPCR, similar to our previous studies using microarray and nCounter data (Corton et al., 2018).

The Running Fisher test was also combined with the three‐pronged approach and the individual analyses (Table 3). Overall calls were determined using the same rule applied in the three‐pronged method (one DDI call indicates overall DDI classification, otherwise non‐DDI). All combinations containing the Running Fisher test produced 100% sensitivity, 81% specificity, and 91% balanced accuracy, suggesting that the classification approaches are complementary to each other and can be applied in conjunction.

Li et al. previously noted some limitations of the biomarker in assessing antimetabolites in both the microarray and nCounter external validation (Li et al., 2017). One of the two reference chemicals misclassified by TGx‐DDI biomarker measured *via* qPCR using the three‐pronged approach was MTX, an antimetabolic DDI agent. In Li et al. (2015), MTX induced smaller fold changes in the biomarker gene expression data, producing weaker signals in the heatmap when compared to other DDI agents in the reference set. In their 2015 paper, Li et al. reported that gene panels smaller than the initial 65‐gene panel misclassified MTX. The qPCR analytical method reduced TGx‐DDI biomarker to a gene set containing 61 genes, due to the exclusion of three genes with low signals. This smaller gene set could be a contributing factor to the misclassification of MTX and other chemicals that induced weaker gene expression changes, such as busulfan and chlorambucil (Fig. 1A,B). Moreover, it has been shown that MTX indirectly inhibits DNA synthesis. MTX targets dihydrofolate reductase (DHFR) to inhibit the conversion of dihydrofolate to tetrahydrofolate (THF), which is the precursor of 5,10‐methylene THF. 5,10‐methylene THF is a cofactor in the thymidylate synthase reaction; the inhibition of DHFR by MTX eventually hinders *de novo* synthesis of thymidine (White and Goldman, 1975; Bender and Makula, 1978). Other antimetabolites in the reference set, such as hydroxyurea and 5‐fluorouracil, inhibit ribonucleotide reductase and thymidylate synthase, respectively, and directly inhibit nucleotide synthesis (Krakoff et al., 1968; Spears et al., 1984). Both were correctly classified (Figs. 1 and 2). It is possible that the 4 h MTX exposure may be insufficient to induce a similar level of DNA damage and subsequent transcriptional responses as other DDI agents that can be measured in the 61‐gene panel.

The DDI grouping includes genotoxicants that directly or indirectly interact with DNA; however, the diverse MOAs within the group are not distinguishable. Moreover, the non‐DDI group contains non‐genotoxic agents as well as aneugens, which cause abnormal chromosome numbers in daughter cells through the interference with the mitotic machinery (Parry et al., 1996). Although considered non‐DDI in this study, aneugens cause genomic damage and, thus, are genotoxicants. Moving forward, investigating other potential signatures specific to MOAs contained in the reference set may be useful for querying a single whole transcriptome expression profile for different genotoxic mechanisms.

Recently, Ates et al. developed a 96‐well qPCR array for analyzing a proprietary gene expression signature of genotoxicity in HepaRG cells (Ates et al., 2018). While the experimental approach is similar to our study, this gene expression signature is analyzed after a 72 h exposure based on a reference set of known *in vivo* genotoxicants that contains aneugens (e.g., vinblastine) that are included in the non‐DDI category of the TGx‐DDI reference set. Thus, positive calls made by the two biomarkers provide different information regarding the chemical's interaction with DNA.

The development and validation of the qPCR analytical methodology for the TGx‐DDI biomarker addresses the challenge that laboratories without whole transcriptome profiling capacity may face in applying TGx‐DDI. Another barrier may be the statistical analyses that are required for classification. Recently, an online chemical classification application was released that uses the original TK6 biomarker developed on Agilent Whole Genome microarrays (URL: https://manticore.niehs.nih.gov/tgclassifier/gthome.php) (Jackson et al., 2017). Users can upload their own transcriptomic data for test chemicals and vehicle controls to this open access tool for the three‐pronged chemical classification; the tool eliminates the need for in‐house biostatistics expertise to use the biomarker. The online tool currently supports Affymetrix Human Genome arrays, Agilent Whole Human Genome arrays, and generic arrays with log_2_ transformed gene fold change values. The qPCR dataset generated in the present study will provide a qPCR‐specific reference expression profile to the online tool for future studies applying the biomarker in the qPCR format.

In summary, we reproduced the TGx‐DDI biomarker using qPCR assays in 96‐well arrays to broaden its accessibility. The utility of the qPCR expression signature as a biomarker to identify DDI chemicals was tested using a set of 24 external chemicals. The qPCR analytical methodology was consistent with the microarray and nCounter methodologies for the TGx‐DDI biomarker, although somewhat less accurate than the latter technologies. The results demonstrate that DDI classifications can be made using this qPCR approach. Furthermore, this work complements the TGx‐DDI online chemical classification tool discussed above; as an addition to the online tool, the qPCR dataset will further facilitate the application of TGx‐DDI and its incorporation in genotoxicity assessment.

### STATEMENT OF AUTHOR CONTRIBUTIONS

CLY, JB, HHL, AF, JA, and EC designed the study. CLY obtained funding to support the project. HHL and RXC conducted the cellular exposures. JB extracted all RNA samples. EC performed the qPCR. AW conducted the statistical analyses and prepared figures. JCC conducted the Running Fisher test and prepared the methods and results sections for this analysis. EC prepared the manuscript with important intellectual input from all authors. All authors had access to the study data and approved the final manuscript.

## Supporting information

Supplementary Table I. **TaqMan Assay ID of 64 TGx‐DDI Biomarker Genes and qPCR Reference Genes**
Supplementary Table II. **Location of Each TaqMan qPCR Assay in the 96‐Well Array**
Supplementary Figure 1. Layout of the custom 96‐well qPCR array. Duplicate assay indicates an alternate TaqMan assay for select biomarker genes. One of the two assays was chosen for the final analyses based on biomarker performance.

Supplementary Fig. 2. **A‐X** Principal Component Analysis (PCA) of the qPCR profiles of the reference set and individual validation agents (left panel) and two‐dimensional clustering (2‐DC) of the qPCR profiles of the reference set and individual validation agents (right panel). Red font indicates DDI reference agents and blue font indicates non‐DDI agents. Green front represents the external validation chemical.
