# Supplementary material for: Assessment of the performance of the TGx‐DDI biomarker to detect DNA damage‐inducing agents using quantitative RT‐PCR in TK6 cells
Source: Environ Mol Mutagen. 2018 Nov 29;60(2):122–33. doi: 10.1002/em.22257 (PMC6588084; doi:10.1002/em.22257)
Supplement: Supplementary file 1 — Supplementary Table I. TaqMan Assay ID of 64 TGx‐DDI Biomarker Genes and qPCR Reference Genes Supplementary Table II. Location of Each TaqMan qPCR Assay in the 96‐Well Array Supplementary Figure 1. Layout of the custom 96‐well qPCR array. Duplicate assay indicates an alternate TaqMan assay for select biomarker genes. One of the two assays was chosen for the final analyses based on biomarker performance. [file EM-60-122-s002.docx]

**SUPPLEMENTARY MATRIAL**

**SUPPLEMENTARY TABLE I. TaqMan Assay ID of 64 TGx-DDI Biomarker Genes and qPCR Reference Genes**

| Gene Symbol | TaqMan Assay ID | Genbank Accession | Entrez Gene ID | Gene Name |
| --- | --- | --- | --- | --- |
| 18S * | Hs99999901_s1 | X03205.1 | HSRRN18s | eukaryotic 18S rRNA |
| ACTA2 | Hs00426835_g1 | NM_001613 | 59 | actin, alpha 2, smooth muscle, aorta |
| ARRDC4 | Hs00411771_m1 | NM_183376 | 91947 | arrestin domain containing 4 |
| B3GNT2 | Hs01935859_s1 | NM_006577 | 10678 | UDP-GlcNAc:betaGal beta-1,3-N-acetylglucosaminyltransferase 2 |
| BLOC1S2 | Hs00611274_m1;  Hs01546558_g1 | NM_001001342 | 282991 | biogenesis of lysosomal organelles complex-1, subunit 2 |
| BRMS1L | Hs00260608_m1 | NM_032352 | 84312 | breast cancer metastasis-suppressor 1-like |
| BTG2 | Hs00198887_m1 | NM_006763 | 7832 | BTG family, member 2 |
| C12orf5 | Hs00608646_m1;  Hs00608644_m1 | NM_020375 | 57103 | chromosome 12 open reading frame 5 |
| CASC3 * | Hs00904843_g1 | NM_007359.4 | 22794 | cancer susceptibility candidate 3 |
| CBLB | Hs00909783_m1 | NM_170662 | 868 | Cas-Br-M (murine) ecotropic retroviral transforming sequence b |
| CDKN1A | Hs00355782_m1;  Hs99999142_m1 | NM_078467 | 1026 | cyclin-dependent kinase inhibitor 1A (p21, Cip1) |
| CEBPD | Hs00270931_s1 | NM_005195 | 1052 | CCAAT/enhancer binding protein (C/EBP), delta |
| CENPE | Hs01068241_m1 | NM_001813 | 1062 | centromere protein E, 312kDa |
| COIL | Hs00982300_m1 | NM_004645 | 8161 | coilin |
| CCP110 (CP110) | Hs00206922_m1;  Hs01015810_g1 | NM_014711 | 9738 | centriolar coiled coil protein 110kDa |
| DAAM1 | Hs00982998_m1 | NM_014992 | 23002 | dishevelled associated activator of morphogenesis 1 |
| DCP1B | Hs00398931_m1;  Hs00398936_m1 | NM_152640 | 196513 | DCP1 decapping enzyme homolog B (S. cerevisiae) |
| DDB2 | Hs03044953_m1;  Hs03044949_m1 | NM_000107 | 1643 | damage-specific DNA binding protein 2, 48kDa |
| DUSP14 | Hs01877076_s1;  Hs00199767_m1 | NM_007026 | 11072 | dual specificity phosphatase 14 |
| E2F7 | Hs00403170_m1;  Hs00987773_m1 | NM_203394 | 144455 | E2F transcription factor 7 |
| E2F8 | Hs00226635_m1;  Hs01079649_m1 | NM_024680 | 79733 | E2F transcription factor 8 |
| EI24 | Hs00903035_g1 | NM_004879 | 9538 | etoposide induced 2.4 mRNA |
| EIF2B1 * | Hs00426752_m1 | NM_001414.3 | 1967 | eukaryotic translation initiation factor 2B subunit alpha |
| FBXO22 | Hs00201796_m1;  Hs00992732_g1 | NM_012170; NM_147188 | 26263 | F-box protein 22 |
| FAM123B (FLJ39827) | Hs01937181_s1 | NM_152424 | 139285 | family with sequence similarity 123B |
| GADD45A | Hs00169255_m1;  Hs99999173_m1 | NM_001924 | 1647 | growth arrest and DNA-damage-inducible, alpha |
| GAPDH * | Hs99999905_m1 | NM_002046.4 | 2597 | glyceraldehyde 3-phosphate dehydrogenase |
| GUSB * | Hs99999908_m1 | NM_00181.3 | 2990 | glucuronidase, beta |
| GXYLT1 (GLT8D3) | Hs01035388_g1 | NM_173601 | 283464 | glucoside xylosyltransferase 1 |
| HIST1H1E | Hs00271195_s1 | NM_005321 | 3008 | histone cluster 1, H1e |
| HIST1H2BB | Hs00606684_s1 | NM_021062 | 3018 | histone cluster 1, H2bb |
| HIST1H2BC | Hs00818513_sH | NM_003526 | 8347 | histone cluster 1, H2bc |
| HIST1H2BG | Hs00374317_s1;  Hs01027934_s1 | NM_003518 | 8339 | histone cluster 1, H2bg |
| HIST1H2BI | Hs01549641_s1 | NM_003525 | 8346 | histone cluster 1, H2bi |
| HIST1H2BM | Hs00605772_s1 | NM_003521 | 8342 | histone cluster 1, H2bm |
| HIST1H2BN | Hs00534080_s1 | NM_003520 | 8341 | histone cluster 1, H2bn |
| HIST1H3D | Hs00371415_s1 | NM_003530 | 8351 | histone cluster 1, H3d |
| HPRT1 * | Hs99999909_m1 | NM_000194.2 | 3251 | Hypoxanthine phosphoribosyltransferase 1 |
| ID2 | Hs04187239_m1 | NM_002166 | 3398 | inhibitor of DNA binding 2, dominant negative helix-loop-helix protein |
| IKBIP (IKIP) | Hs01096287_m1 | NM_153687;  NM_201612 | 121457 | IKBKB interacting protein |
| AEN (ISG20L1) | Hs00901422_m1;  Hs00224322_m1 | NM_022767 | 64782 | apoptosis enhancing nuclease |
| ITPKC | Hs00363893_m1 | NM_025194 | 80271 | inositol-trisphosphate 3-kinase C |
| ITPR1 | Hs00181881_m1 | NM_002222 | 3708 | inositol 1,4,5-trisphosphate receptor, type 1 |
| LCE1E | Hs02380094_g1;  Hs02573836_s1 | NM_178353 | 353135 | late cornified envelope 1E |
| LRRFIP2 | Hs00992891_g1 | NM_006309; NM_017724 | 9209 | leucine rich repeat (in FLII) interacting protein 2 |
| MDM2 | Hs01066930_m1;  Hs00540450_s1 | NM_002392 | 4193 | Mdm2 p53 binding protein homolog (mouse) |
| NLRX1 (NOD9) | Hs01123236_m1 | NM_170722 | 79671 | NLR family member X1 |
| PCDH8 | Hs04187285_g1 | NM_002590 | 5100 | protocadherin 8 |
| PHLDA3 | Hs00385313_m1;  Hs00247337_m1 | NM_012396 | 23612 | pleckstrin homology-like domain, family A, member 3 |
| PLK3 | Hs00177725_m1 | NM_004073 | 1263 | polo-like kinase 3 |
| PPM1D | Hs01013292_m1;  Hs00186230_m1 | NM_003620 | 8493 | protein phosphatase, Mg2+/Mn2+ dependent, 1D |
| PRKAB1 | Hs00272166_m1;  Hs01058464_g1 | NM_006253 | 5564 | protein kinase, AMP-activated, beta 1 non-catalytic subunit |
| PRKAB2 | Hs00975854_g1 | NM_005399 | 5565 | protein kinase, AMP-activated, beta 2 non-catalytic subunit |
| PTGER4 | Hs00168761_m1 | NM_000958 | 5734 | prostaglandin E receptor 4 (subtype EP4) |
| RAPGEF2 | Hs01127851_m1 | NM_014247 | 9693 | Rap guanine nucleotide exchange factor (GEF) 2 |
| RBM12B | Hs01941687_s1 | NM_203390 | 389677 | RNA binding motif protein 12B |
| MEX3B (RKHD3) | Hs00863082_m1 | NM_032246 | 84206 | mex-3 homolog B (C. elegans) |
| RPS27L | Hs00955038_g1 | NM_015920 | 51065 | ribosomal protein S27-like |
| RRM2B | Hs00968432_m1 | NM_015713 | 50484 | ribonucleotide reductase M2 B (TP53 inducible) |
| SEL1L | Hs01071406_m1 | AB020335; NM_005065 | 6400 | sel-1 suppressor of lin-12-like (C. elegans) |
| SEMG2 | Hs03044701_m1 | NM_003008 | 6407 | semenogelin II |
| SERTAD1 | Hs01932680_s1;  Hs01932680_s1 | NM_013376 | 29950 | SERTA domain containing 1 |
| SMAD5 | Hs00195437_m1 | NM_001001419 | 4090 | SMAD family member 5 |
| TM7SF3 | Hs00213081_m1 | NM_016551 | 51768 | transmembrane 7 superfamily member 3 |
| TNFRSF17 | Hs00171292_m1 | NM_001192 | 608 | tumor necrosis factor receptor superfamily, member 17 |
| TOPORS | Hs00359341_m1;  Hs01557143_m1 | NM_005802 | 10210 | topoisomerase I binding, arginine/serine-rich, E3 ubiquitin protein ligase |
| TP53I3 | Hs00936520_m1 | NM_004881 | 9540 | tumor protein p53 inducible protein 3 |
| TRIAP1 | Hs00429934_g1 | NM_016399 | 51499 | TP53 regulated inhibitor of apoptosis 1 |
| TRIM22 | Hs01001179_m1;  Hs01001178_m1 | NM_006074 | 10346 | tripartite motif containing 22 |
| USP41 | Hs02596851_gH | ENST00000454608 (XM_036729) | 373856 | ubiquitin specific peptidase 41 |

The 64-gene signature developed by Li et al. [2015] [Li et al., 2015]. Previous gene names or gene aliases are shown in brackets in the Gene Symbol column. Asterisk (*) indicates qPCR reference genes for normalization.

**SUPPLEMENTARY TABLE II.** **Location of Each TaqMan qPCR Assay in the 96-Well Array**

| Row | Column | TaqMan Assay ID | Gene |
| --- | --- | --- | --- |
| A | 1 | Preloaded Reference gene | *18s** |
| A | 2 | Preloaded Reference gene | *GAPDH** |
| A | 3 | Preloaded Reference gene | *HPRT** |
| A | 4 | Preloaded Reference gene | *GUSB** |
| A | 5 | Hs00426752_m1 | *EIF2B1** |
| A | 6 | Hs00904843_g1 | *CASC3** |
| A | 7 | Hs00186230_m1 | *PPM1D* |
| A | 8 | Hs01058464_g1 | *PRKAB1* |
| A | 9 | Hs00247337_m1 | *PHLDA3* |
| A | 10 | Hs00987773_m1 | *E2F7* |
| A | 11 | Hs01932680_s1 | *SERTAD1* |
| A | 12 | Hs00429934_g1 | *TRIAP1* |
| B | 1 | Hs01546558_g1 | *BLOC1S2* |
| B | 2 | Hs00426835_g1 | *ACTA2* |
| B | 3 | Hs00411771_m1 | *ARRDC4* |
| B | 4 | Hs01935859_s1 | *B3GNT2* |
| B | 5 | Hs00611274_m1 | *BLOC1S2* |
| B | 6 | Hs00260608_m1 | *BRMS1L* |
| B | 7 | Hs00198887_m1 | *BTG2* |
| B | 8 | Hs00608646_m1 | *C12orf5* |
| B | 9 | Hs00909783_m1 | *CBLB* |
| B | 10 | Hs00355782_m1 | *CDKN1A* |
| B | 11 | Hs00270931_s1 | *CEBPD* |
| B | 12 | Hs00224322_m1 | *AEN* |
| C | 1 | Hs03044949_m1 | *DDB2* |
| C | 2 | Hs01068241_m1 | *CENPE* |
| C | 3 | Hs00982300_m1 | *COIL* |
| C | 4 | Hs00206922_m1 | *CCP110 (CP110)* |
| C | 5 | Hs00982998_m1 | *DAAM1* |
| C | 6 | Hs00398931_m1 | *DCP1B* |
| C | 7 | Hs03044953_m1 | *DDB2* |
| C | 8 | Hs01877076_s1 | *DUSP14* |
| C | 9 | Hs00403170_m1 | *E2F7* |
| C | 10 | Hs00226635_m1 | *E2F8* |
| C | 11 | Hs00903035_g1 | *EI24* |
| C | 12 | Hs00198887_m1 | *BTG2* |
| D | 1 | Hs01001178_m1 | *TRIM22* |
| D | 2 | Hs00201796_m1 | *FBXO22* |
| D | 3 | Hs01937181_s1 | *FAM123B/ AMER1* |
| D | 4 | Hs00169255_m1 | *GADD45A* |
| D | 5 | Hs01035388_g1 | *GXYLT1* |
| D | 6 | Hs00271195_s1 | *HIST1H1E* |
| D | 7 | Hs00606684_s1 | *HIST1H2BB* |
| D | 8 | Hs00818513_sH | *HIST1H2BC* |
| D | 9 | Hs00374317_s1 | *HIST1H2BG* |
| D | 10 | Hs01549641_s1 | *HIST1H2BI* |
| D | 11 | Hs00605772_s1 | *HIST1H2BM* |
| D | 12 | Hs04187285_g1 | *PCDH8* |
| E | 1 | Hs00540450_s1 | *MDM2* |
| E | 2 | Hs00534080_s1 | *HIST1H2BN* |
| E | 3 | Hs00371415_s1 | *HIST1H3D* |
| E | 4 | Hs04187239_m1 | *ID2* |
| E | 5 | Hs01096287_m1 | *IKBIP* |
| E | 6 | Hs00901422_m1 | *AEN* |
| E | 7 | Hs00363893_m1 | *ITPKC* |
| E | 8 | Hs00181881_m1 | *ITPR1* |
| E | 9 | Hs02380094_g1 | *LCE1E* |
| E | 10 | Hs00992891_g1 | *LRRFIP2* |
| E | 11 | Hs01066930_m1 | *MDM2* |
| E | 12 | Hs00398936_m1 | *DCP1B* |
| F | 1 | Hs00936520_m1 | *TP53I3* |
| F | 2 | Hs01123236_m1 | *NLRX1* |
| F | 3 | Hs04187285_g1 | *PCDH8* |
| F | 4 | Hs00385313_m1 | *PHLDA3* |
| F | 5 | Hs00177725_m1 | *PLK3* |
| F | 6 | Hs01013292_m1 | *PPM1D* |
| F | 7 | Hs00272166_m1 | *PRKAB1* |
| F | 8 | Hs00975854_g1 | *PRKAB2* |
| F | 9 | Hs00168761_m1 | *PTGER4* |
| F | 10 | Hs01127851_m1 | *RAPGEF2* |
| F | 11 | Hs01941687_s1 | *RBM12B* |
| F | 12 | Hs00992732_g1 | *FBXO22* |
| G | 1 | Hs99999142_m1 | *CDKN1A* |
| G | 2 | Hs00863082_m1 | *MEX3B* |
| G | 3 | Hs00955038_g1 | *RPS27L* |
| G | 4 | Hs00968432_m1 | *RRM2B* |
| G | 5 | Hs01071406_m1 | *SEL1L* |
| G | 6 | Hs03044701_m1 | *SEMG2* |
| G | 7 | Hs01932680_s1 | *SERTAD1* |
| G | 8 | Hs00195437_m1 | *SMAD5* |
| G | 9 | Hs00213081_m1 | *TM7SF3* |
| G | 10 | Hs00171292_m1 | *TNFRSF17* |
| G | 11 | Hs00359341_m1 | *TOPORS* |
| G | 12 | Hs02573836_s1 | *LCE1E* |
| H | 1 | Hs01079649_m1 | *E2F8* |
| H | 2 | Hs00936520_m1 | *TP53I3* |
| H | 3 | Hs00429934_g1 | *TRIAP1* |
| H | 4 | Hs01001179_m1 | *TRIM22* |
| H | 5 | Hs02596851_gH | *USP41* |
| H | 6 | Hs01015810_g1 | *CCP110 (CP110)* |
| H | 7 | Hs99999173_m1 | *GADD45A* |
| H | 8 | Hs00608644_m1 | *C12orf5* |
| H | 9 | Hs00199767_m1 | *DUSP14* |
| H | 10 | Hs01557143_m1 | *TOPORS* |
| H | 11 | Hs01027934_s1 | *HIST1H2BG* |
| H | 12 | Hs02596851_gH | *USP41* |

Highlighted assays/genes indicate the assays that were included in the final analyses. The TaqMan assays were chosen based on the biomarker performance.

* indicates reference genes

The ‘TaqMan Array Plate 96 Plus’ format contains four preloaded assays for reference genes (18s, GAPDH, HPRT, and GUSB) (Applied Biosystems, Burlington, ON, Canada)

**Supplementary Fig. 1.** Layout of the custom 96-well qPCR array. Duplicate assay indicates an alternate TaqMan assay for select biomarker genes. One of the two assays was chosen for the final analyses based on biomarker performance.

**Supplementary Fig. 2. A-X** Principal Component Analysis (PCA) of the qPCR profiles of the reference set and individual validation agents (left panel) and two-dimensional clustering (2-DC) of the qPCR profiles of the reference set and individual validation agents (right panel). Red font indicates DDI reference agents and blue font indicates non-DDI agents. Green front represents the external validation chemical.
