# Supplementary material for: Assessment of the performance of the TGx‐DDI biomarker to detect DNA damage‐inducing agents using quantitative RT‐PCR in TK6 cells
Source: Environ Mol Mutagen. 2018 Nov 29;60(2):122–33. doi: 10.1002/em.22257 (PMC6588084; doi:10.1002/em.22257)
Supplement: Supplementary file 2 — Supplementary Fig. 2. A‐X Principal Component Analysis (PCA) of the qPCR profiles of the reference set and individual validation agents (left panel) and two‐dimensional clustering (2‐DC) of the qPCR profiles of the reference set and individual validation agents (right panel). Red font indicates DDI reference agents and blue font indicates non‐DDI agents. Green front represents the external validation chemical. [file EM-60-122-s001.docx]

# A

MMS

Cisplatin


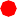

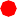

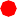

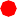

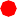

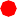

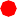

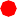

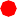

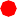

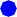

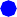

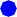

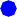

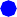

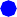

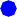

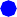

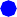

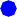

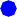

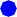

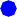

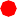

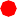


Methotrexate

Thapsigargin

CdCl2

Vinblastine

Heat Shock (47oC) Paclitaxel Trichostatin A

Colchicine

HC Toxin

OxamflatinApicidin

Docetaxel

4% EtOH

2% EtOH

Tunicamycin

Antimycin A

MMS

Cisplatin

Camptothecin 5-FU

Hydroxyurea

Ara C

Etoposide

H2O2

NaAsO2

4 Gy - Gamma ray Bleomycin

K2CrO4

Bleomycin

2-DG

Etoposide

5-FU

10

4 Gy - Gamma ray

Bleomycin

Camptothecin

NaAsO2

H2O2

Bleomycin 2-DG K2CrO4

5

Paclitaxel

Docetaxel Trichostatin A

0

Heat Shock (47o C) Vinblastine Colchicine Methotrexate Thapsigargin

CdCl2 Tunicamycin Antimycin A

-5

4% EtOH

2% EtOH

Oxamflatin

Apicidin

-10

HC Toxin

PC 2

## -10 -5 0 5 10

Hydroxyurea Ara C

# B

Paclitaxel

Docetaxel


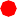

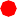

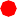

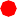

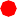

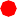

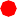

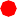

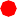

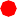

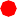

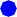

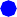

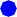

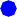

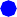

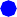

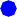

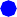

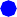

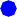

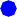

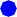

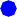

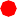

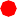


Methotrexate

Thapsigargin

CdCl2

Vinblastine

Heat Shock (47oC) Paclitaxel Trichostatin A

Colchicine

HC Toxin

OxamflatinApicidin

Busulfan

Docetaxel

4% EtOH

2% EtOH

Tunicamycin

Antimycin A

MMS

Cisplatin

Camptothecin 5-FU

Hydroxyurea

Ara C

Etoposide

H2O2

NaAsO2

4 Gy - Gamma ray

K2CrO4

Bleomycin

2-DG

Trichostatin A

Heat Shock (47o C)

10

Vinblastine

Colchicine

Methotrexate

Thapsigargin

CdCl2

Tunicamycin Antimycin A 4% EtOH

5

2% EtOH

Oxamflatin Apicidin HC Toxin Busulfan NaAsO2 MMS

0

Cisplatin

Etoposide 5-FU

-5

4 Gy - Gamma ray

Camptothecin

H2O2

Bleomycin

2-DG

-10

K2CrO4

PC 2

## -10 -5 0 5 10

Hydroxyurea Ara C

MMS

Cisplatin


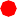

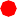

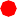

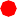

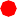

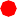

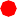

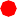

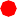

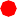

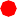

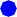

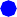

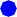

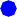

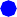

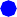

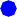

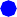

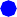

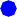

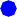

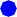

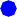

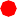

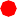


Methotrexate

Chlorambucil

Thapsigargin

CdCl2

Vinblastine

Heat Shock (47oC) Paclitaxel Trichostatin A

Colchicine

HC Toxin

OxamflatinApicidin

Docetaxel

4% EtOH

2% EtOH

Tunicamycin

Antimycin A

MMS

Cisplatin

Camptothecin 5-FU

Hydroxyurea

Ara C

Etoposide

H2O2

NaAsO2

4 Gy - Gamma ray

K2CrO4

Bleomycin

2-DG

Etoposide

5-FU

10

4 Gy - Gamma ray

Camptothecin

H2O2

Bleomycin

2-DG

K2CrO4

5

NaAsO2 Paclitaxel

Docetaxel

Trichostatin A

Heat Shock (47o C) Vinblastin Colchicine Methotrexate Thapsigargin CdCl2

0

Chlorambucil Oxamflatin Apicidin

-5

HC Toxin

Tunicamycin

Antimycin A

4% EtOH

-10

2% EtOH

PC 2

## -10 -5 0 5 10

Hydroxyurea Ara C

# D

MMS

Cisplatin


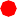

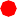

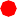

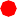

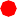

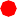

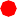

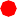

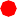

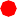

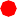

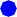

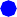

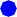

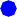

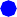

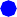

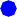

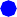

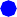

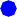

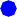

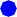


Methotrexate

Thapsigargin

CdCl2

Vinblastine

Heat Shock (47oC) Paclitaxel Trichostatin A

Colchicine

HC Toxin

OxamflatinApicidin

Docetaxel

4% EtOH

2% EtOH

Tunicamycin

Antimycin A

MMS

Cisplatin

Camptothecin 5-FU

Hydroxyurea

Ara C

Etoposide

H2O2

4 Gy - Gamma ray

NaAsO2

EMS

K2CrO4

Bleomycin

2-DG

Etoposide

5-FU

10

4 Gy - Gamma ray

EMS

Camptothecin

NaAsO2

H2O2

Bleomycin 2-DG K2CrO4

5

Paclitaxel

Docetaxel Trichostatin A

0

Heat Shock (47o C) Vinblastin Colchicine Methotrexate Thapsigargin

CdCl2

-5

Tunicamycin Antimycin A

4% EtOH

2% EtOH

Oxamflatin

Apicidin

-10

HC Toxin

PC 2

## -10 -5 0 5 10

Hydroxyurea Ara C

# E

Etoposide

5-FU

Methotrexate

Thapsigargin

CdCl2

Vinblastine

Heat Shock (47oC) Paclitaxel Trichostatin A

Colchicine

HC Toxin

OxamflatinApicidin

Docetaxel

4% EtOH

2% EtOH

Tunicamycin

Antimycin A

MMS

Cisplatin

ENU

5-FU

Camptothecin

Hydroxyurea

Ara C

Etoposide

H2O2

NaAsO2

4 Gy - Gamma ray

K2CrO4

Bleomycin

2-DG

4 Gy - Gamma ray

ENU

10

MMS

Cisplatin

Camptothecin

H2O2

Bleomycin

2-DG K2CrO4

5

NaAsO2

Paclitaxel

Docetaxel Trichostatin A

0

Heat Shock (47o C) Vinblastin Colchicine Methotrexate Thapsigargin

CdCl2 Tunicamycin Antimycin A

-5

4% EtOH

2% EtOH

Oxamflatin

Apicidin

-10

HC Toxin

PC 2

## -10 -5 0 5 10

Hydroxyurea Ara C

# F

MMS

Cisplatin

Methotrexate

Thapsigargin

CdCl2

Vinblastine

Heat Shock (47oC) Paclitaxel Trichostatin A

Colchicine

HC Toxin

OxamflatinApicidin

Docetaxel

4% EtOH

2% EtOH

Tunicamycin

Antimycin A

MMS

Cisplatin

Hydroxyurea

Ara C Camptothecin 5-FU Hydroquinone

Etoposide

H2O2

NaAsO2

4 Gy - Gamma ray

K2CrO4

Bleomycin

2-DG

Etoposide

5-FU

10

4 Gy - Gamma ray

Camptothecin

Hydroquinone

NaAsO2

H2O2

Bleomycin 2-DG K2CrO4

5

Paclitaxel

Docetaxel Trichostatin A

0

Heat Shock (47o C) Vinblastin Colchicine Methotrexate Thapsigargin

CdCl2 Tunicamycin Antimycin A

-5

4% EtOH

2% EtOH

Oxamflatin

Apicidin

-10

HC Toxin

PC 2

## -10 -5 0 5 10

Hydroxyurea Ara C

# G

MMS

Cisplatin

Methotrexate

Thapsigargin

CdCl2

Vinblastine

Heat Shock (47oC) Paclitaxel Trichostatin A

Colchicine

HC Toxin

OxamflatinApicidin

Docetaxel

4% EtOH

2% EtOH

Tunicamycin

Antimycin A

MMS

Cisplatin

Camptothecin 5-FU

Hydroxyurea

Ara C

Etoposide

H2O2

NaAsO2

Mytomycin C

4 Gy - Gamma ray

K2CrO4

Bleomycin

2-DG

Etoposide

5-FU

10

4 Gy - Gamma ray

Mytomycin C

Camptothecin

NaAsO2

H2O2

Bleomycin 2-DG K2CrO4

5

Paclitaxel Docetaxel Trichostatin A

0

Heat Shock (47o C) Vinblastin Colchicine Methotrexate Thapsigargin

CdCl2 Tunicamycin Antimycin A

-5

4% EtOH

2% EtOH

Oxamflatin

Apicidin

-10

HC Toxin

PC 2

## -10 -5 0 5 10

Hydroxyurea Ara C

# H

MMS

Cisplatin

Methotrexate

Thapsigargin

CdCl2

Vinblastine

Heat Shock (47oC) Paclitaxel Trichostatin A

Colchicine

HC Toxin

OxamflatinApicidin

Docetaxel

4% EtOH

2% EtOH

Tunicamycin

Antimycin A

MMS

Cisplatin

Nitrogen Mustard

Camptothecin 5-FU

Hydroxyurea

Ara C

Etoposide

H2O2

NaAsO2

4 Gy - Gamma ray

K2CrO4

Bleomycin

2-DG

Nitrogen Mustard

Etoposide

10

5-FU

4 Gy - Gamma ray

Camptothecin

NaAsO2

H2O2

Bleomycin 2-DG K2CrO4

5

Paclitaxel

Docetaxel Trichostatin A

0

Heat Shock (47o C) Vinblastin Colchicine Methotrexate Thapsigargin

CdCl2 Tunicamycin Antimycin A

-5

4% EtOH

2% EtOH

Oxamflatin

Apicidin

-10

HC Toxin

PC 2

## -10 -5 0 5 10

Hydroxyurea Ara C

# I

MMS

Cisplatin

Methotrexate

Thapsigargin

CdCl2

Vinblastine

Heat Shock (47oC) Paclitaxel Trichostatin A

Colchicine

HC Toxin

OxamflatinApicidin

Docetaxel

4% EtOH

2% EtOH

Tunicamycin

Ampicilin

Antimycin A

MMS

Cisplatin

Camptothecin 5-FU

Hydroxyurea

Ara C

Etoposide

H2O2

NaAsO2

4 Gy - Gamma ray

K2CrO4

Bleomycin

2-DG

Etoposide

5-FU

10

4 Gy - Gamma ray

Camptothecin

H2O2

Bleomycin

2-DG

K2CrO4

5

NaAsO2 Paclitaxel

Docetaxel

Trichostatin A Heat Shock (47 C) Vinblastin Colchicine Methotrexate Thapsigargin CdCl2

0

Tunicamycin Antimycin A 4% EtOH

-5

2% EtOH

Oxamflatin

Apicidin

HC Toxin

-10

Ampicilin

PC 2

## -10 -5 0 5 10

Hydroxyurea Ara C

# J

MMS

Cisplatin

Methotrexate

Thapsigargin

CdCl2

Vinblastine

Heat Shock (47oC) Paclitaxel Trichostatin A

Colchicine

OxamflatinApicidin

Docetaxel

Erythromycin

HC Toxin

4% EtOH

2% EtOH

Tunicamycin

Antimycin A

MMS

Cisplatin

Camptothecin 5-FU

Hydroxyurea

Ara C

Etoposide

H2O2

NaAsO2

4 Gy - Gamma ray

K2CrO4

Bleomycin

2-DG

Etoposide

5-FU

10

4 Gy - Gamma ray

Camptothecin

H2O2

Bleomycin

2-DG

K2CrO4

5

NaAsO2 Paclitaxel

Docetaxel

Trichostatin A

Heat Shock (47 C)

Vinblastin Colchicine Methotrexate Thapsigargin Erythromycin

0

CdCl2 Tunicamycin Antimycin A

-5

4% EtOH

2% EtOH

Oxamflatin

Apicidin

-10

HC Toxin

PC 2

## -10 -5 0 5 10

Hydroxyurea Ara C

# K

Paclitaxel

Docetaxel

Methotrexate

Thapsigargin

CdCl2

Vinblastine

Heat Shock (47oC) Paclitaxel Trichostatin A

Colchicine

OxamflatinApicidin

Docetaxel

HC Toxin

4% EtOH

2% EtOH

Antimycin A Methyl carbamate

Tunicamycin

MMS

Cisplatin

Camptothecin 5-FU

Hydroxyurea

Ara C

Etoposide

H2O2

NaAsO2

4 Gy - Gamma ray

K2CrO4

Bleomycin

2-DG

Trichostatin A

Heat Shock (47 C)

10

Vinblastin

Colchicine

Methotrexate

Thapsigargin

CdCl2

Tunicamycin Antimycin A 4% EtOH

5

2% EtOH

Oxamflatin

Apicidin

HC Toxin

0

Methyl carbamate MMS

Cisplatin Etoposide

5-FU

-5

4 Gy - Gamma ray Camptothecin

H2O2

Bleomycin

2-DG

K2CrO4

-10

NaAsO2

PC 2

## -10 -5 0 5 10

Hydroxyurea Ara C

Paclitaxel

Docetaxel

Methotrexate

Thapsigargin

CdCl2

Vinblastine

Heat Shock (47oC) Paclitaxel Trichostatin A

Colchicine

OxamflatinApicidin

Docetaxel

HC Toxin

4% EtOH

2% EtOH

Tunicamycin

N butyl Chloride

Antimycin A

MMS

Cisplatin

Camptothecin 5-FU

Hydroxyurea

Ara C

Etoposide

H2O2

NaAsO2

4 Gy - Gamma ray

K2CrO4

Bleomycin

2-DG

Trichostatin A

Heat Shock (47 C)

10

Vinblastin

Colchicine

Methotrexate

Thapsigargin

CdCl2

Tunicamycin Antimycin A 4% EtOH

5

2% EtOH

Oxamflatin

Apicidin

HC Toxin

0

N butyl Chloride MMS

Cisplatin Etoposide

5-FU

-5

4 Gy - Gamma ray Camptothecin

H2O2

Bleomycin

2-DG

K2CrO4

-10

NaAsO2

PC 2

## -10 -5 0 5 10

Hydroxyurea Ara C

# M

Sunitibib malate

NaAsO2

Methotrexate

Thapsigargin

CdCl2

Vinblastine

Heat Shock (47oC) Paclitaxel Trichostatin A

Colchicine

OxamflatinApicidin

Docetaxel

HC Toxin

4% EtOH

2% EtOH

Tunicamycin

Antimycin A

MMS

Cisplatin

Camptothecin 5-FU

Hydroxyurea

Ara C

Etoposide

H2O2

NaAsO2 Sunitibib malate

4 Gy - Gamma ray

K2CrO4

Bleomycin

2-DG

MMS

Cisplatin

10

Etoposide

5-FU

4 Gy - Gamma ray

Camptothecin

H2O2

Bleomycin 2-DG K2CrO4

5

Paclitaxel

Docetaxel

Trichostatin A

Heat Shock (47 C) Vinblastin Colchicine Methotrexate Thapsigargin

0

CdCl2 Tunicamycin Antimycin A

-5

4% EtOH

2% EtOH

Oxamflatin

Apicidin

-10

HC Toxin

PC 2

## -10 -5 0 5 10

Hydroxyurea Ara C

# N

MMS

Cisplatin

Methotrexate

Thapsigargin

CdCl2

Vinblastine

Heat Shock (47oC) Paclitaxel Trichostatin A

Colchicine

OxamflatinApicidin

Docetaxel

HC Toxin

Tunicamycin

2, 4 DNP

4% EtOH

2% EtOH

Antimycin A

MMS

Cisplatin

Camptothecin 5-FU

Hydroxyurea

Ara C

Etoposide

H2O2

NaAsO2

4 Gy - Gamma ray

K2CrO4

Bleomycin

2-DG

Etoposide

5-FU

10

4 Gy - Gamma ray

Camptothecin

H2O2

Bleomycin

2-DG

K2CrO4

5

NaAsO2 Paclitaxel

Docetaxel

Trichostatin A

Heat Shock (47 C)

Vinblastin Colchicine Methotrexate Thapsigargin CdCl2

0

Tunicamycin Antimycin A 2,4 DNP

-5

4% EtOH

2% EtOH

Oxamflatin

Apicidin

-10

HC Toxin

PC 2

## -10 -5 0 5 10

Hydroxyurea Ara C

# O

MMS

Cisplatin

Methotrexate

Thapsigargin

CdCl2

Vinblastine

Heat Shock (47oC) Paclitaxel Trichostatin A

Colchicine

OxamflatinApicidin

Docetaxel

HC Toxin

4% EtOH

2% EtOH

Tunicamycin

Antimycin A

MMS

Cisplatin

Camptothecin 5-FU

Hydroxyurea

Ara C

Etoposide

H2O2

NaAsO2

4 Gy - Gamma ray

K2CrO4

Bleomycin

2-DG

Cycloheximide

Etoposide

5-FU

10

4 Gy - Gamma ray

Camptothecin

H2O2

Bleomycin

2-DG

K2CrO4

5

NaAsO2 Paclitaxel

Docetaxel

Trichostatin A

Heat Shock (47 C)

Vinblastin Colchicine Methotrexate Thapsigargin CdCl2

0

Tunicamycin Antimycin A 4% EtOH

-5

2% EtOH

Oxamflatin

Apicidin

HC Toxin

-10

Cycloheximide

PC 2

## -10 -5 0 5 10

Hydroxyurea Ara C

# P

MMS

Cisplatin

Methotrexate

Thapsigargin

CdCl2

Dexamethasone

Vinblastine

Heat Shock (47oC) Paclitaxel Trichostatin A

Colchicine

OxamflatinApicidin

Docetaxel

HC Toxin

4% EtOH

2% EtOH

Tunicamycin

Antimycin A

MMS

Cisplatin

Camptothecin 5-FU

Hydroxyurea

Ara C

Etoposide

H2O2

NaAsO2

4 Gy - Gamma ray

K2CrO4

Bleomycin

2-DG

Etoposide

5-FU

10

4 Gy - Gamma ray

Camptothecin

H2O2

Bleomycin

2-DG

K2CrO_4_

5

NaAsO_2_ Dexamethasone

Thapsigargin

Paclitaxel

Docetaxel

Trichostatin A Heat Shock (47 C) Vinblastin Colchicine Methotrexate

0

CdCl2 Tunicamycin Antimycin A

-5

4% EtOH

2% EtOH

Oxamflatin

Apicidin

-10

HC Toxin

PC 2

## -10 -5 0 5 10

Hydroxyurea Ara C

# Q

Paclitaxel

Docetaxel

Methotrexate

Thapsigargin

CdCl2

Vinblastine

Heat Shock (47oC) Paclitaxel Trichostatin A

Colchicine

Donepezil

HC Toxin

OxamflatinApicidin

Docetaxel

4% EtOH

2% EtOH

Tunicamycin

Antimycin A

MMS

Cisplatin

Camptothecin 5-FU

Hydroxyurea

Ara C

Etoposide

H2O2

NaAsO2

4 Gy - Gamma ray

K2CrO4

Bleomycin

2-DG

Trichostatin A

Heat Shock (47 C)

10

Vinblastin

Colchicine

Methotrexate

Thapsigargin

CdCl2

Tunicamycin Antimycin A 4% EtOH

5

2% EtOH

Oxamflatin

Apicidin

HC Toxin Donepezil MMS

0

Cisplatin Etoposide

5-FU

-5

4 Gy - Gamma ray Camptothecin

H2O2

Bleomycin

2-DG

K2CrO4

-10

NaAsO2

PC 2

## -10 -5 0 5 10

Hydroxyurea Ara C

# R

MMS

Cisplatin

Methotrexate

Thapsigargin

CdCl2

Esomeprazole

Vinblastine

Heat Shock (47oC) Paclitaxel Trichostatin A

Colchicine

HC Toxin

OxamflatinApicidin

Docetaxel

4% EtOH

2% EtOH

Tunicamycin

Antimycin A

MMS

Cisplatin

Camptothecin 5-FU

Hydroxyurea

Ara C

Etoposide

H2O2

NaAsO2

4 Gy - Gamma ray

K2CrO4

Bleomycin

2-DG

Etoposide

5-FU

10

4 Gy - Gamma ray

Camptothecin

H2O2

Bleomycin

2-DG

K2CrO4

5

NaAsO2 Paclitaxel

Docetaxel

Trichostatin A

Heat Shock (47 C)

Vinblastin Colchicine Methotrexate Thapsigargin CdCl2

0

Tunicamycin Antimycin A 4% EtOH

-5

2% EtOH

Oxamflatin

Apicidin

HC Toxin

-10

Esomeprazole

PC 2

## -10 -5 0 5 10

Hydroxyurea Ara C

# S

MMS

Cisplatin

Methotrexate

Thapsigargin

CdCl2

Vinblastine

Heat Shock (47oC) Paclitaxel Trichostatin A

Colchicine

HC Toxin

OxamflatinApicidin

Docetaxel

4% EtOH

2% EtOH

Tunicamycin

Antimycin A

MMS

Exemestane

Cisplatin

Camptothecin 5-FU

Hydroxyurea

Ara C

Etoposide

H2O2

NaAsO2

4 Gy - Gamma ray

K2CrO4

Bleomycin

2-DG

Etoposide

5-FU

10

4 Gy - Gamma ray

Camptothecin

H2O2

Bleomycin

2-DG

K2CrO4

5

NaAsO2 Paclitaxel

Docetaxel

Trichostatin A

Heat Shock (47 C)

Vinblastin Colchicine Methotrexate Thapsigargin CdCl2

0

Tunicamycin Antimycin A 4% EtOH

-5

2% EtOH

Oxamflatin

Apicidin

HC Toxin

-10

Hydroxyurea

PC 2

## -10 -5 0 5 10

Ara C Exemestane

# T

MMS

Cisplatin

Methotrexate

Thapsigargin

CdCl2

Vinblastine

OxamflatinApicidin

Heat Shock (47oC) Paclitaxel Trichostatin A

Colchicine

HC Toxin

Olmesartan Docetaxel

4% EtOH

2% EtOH

Tunicamycin

Antimycin A

MMS

Cisplatin

Camptothecin 5-FU

Hydroxyurea

Ara C

Etoposide

H2O2

NaAsO2

4 Gy - Gamma ray

K2CrO4

Bleomycin

2-DG

Etoposide

5-FU

10

4 Gy - Gamma ray

Camptothecin

H2O2

Bleomycin

2-DG

K2CrO4

5

NaAsO2 Paclitaxel

Docetaxel

Trichostatin A Heat Shock (47 C) Olmesartan Vinblastin Colchicine Methotrexate Thapsigargin

0

CdCl2 Tunicamycin Antimycin A

-5

4% EtOH

2% EtOH

Oxamflatin

Apicidin

-10

HC Toxin

PC 2

## -10 -5 0 5 10

Hydroxyurea Ara C

# U

MMS

Cisplatin

Methotrexate

Thapsigargin

CdCl2

Vinblastine

Heat Shock (47oC) Paclitaxel Trichostatin A

Colchicine

OxamflatinApicidin

Docetaxel

HC Toxin

Phenobarbital

4% EtOH

2% EtOH

Tunicamycin

Antimycin A

MMS

Cisplatin

Camptothecin 5-FU

Hydroxyurea

Ara C

Etoposide

H2O2

NaAsO2

4 Gy - Gamma ray

K2CrO4

Bleomycin

2-DG

Etoposide

5-FU

10

4 Gy - Gamma ray

Camptothecin

H2O2

Bleomycin

2-DG

K2CrO4

5

NaAsO2 Paclitaxel

Docetaxel

Trichostatin A Heat Shock (47 C)

Vinblastin Colchicine Phenobarbital Methotrexate Thapsigargin

0

CdCl2 Tunicamycin Antimycin A

-5

4% EtOH

2% EtOH

Oxamflatin

Apicidin

-10

HC Toxin

PC 2

## -10 -5 0 5 10

Hydroxyurea Ara C

# V

MMS

Cisplatin

Methotrexate

Thapsigargin

CdCl2

Vinblastine

Heat Shock (47oC) Paclitaxel Trichostatin A

Colchicine

HC Toxin

OxamflatinApicidin

Docetaxel

4% EtOH

2% EtOH

Antimycin A

Rabeprazole

Tunicamycin

MMS

Cisplatin

Camptothecin 5-FU

Hydroxyurea

Ara C

Etoposide

H2O2

NaAsO2

4 Gy - Gamma ray

K2CrO4

Bleomycin

2-DG

Etoposide

5-FU

10

4 Gy - Gamma ray

Camptothecin

H2O2

Bleomycin

2-DG

K2CrO4

5

NaAsO2 Paclitaxel

Docetaxel

Trichostatin A

Heat Shock (47 C)

Vinblastin Colchicine Methotrexate Thapsigargin CdCl2

0

Oxamflatin Apicidin HC Toxin

-5

Tunicamycin

Antimycin A

Rabeprazole

4% EtOH

-10

2% EtOH

PC 2

## -10 -5 0 5 10

Hydroxyurea Ara C

# W

MMS

Cisplatin

Methotrexate

Thapsigargin

CdCl2

Vinblastine

Heat Shock (47oC) Paclitaxel Trichostatin A

Colchicine

HC Toxin

OxamflatinApicidin

Docetaxel

Rotigotine

4% EtOH

2% EtOH

Antimycin A

Tunicamycin

MMS

Cisplatin

Camptothecin 5-FU

Hydroxyurea

Ara C

Etoposide

H2O2

NaAsO2

4 Gy - Gamma ray

K2CrO4

Bleomycin

2-DG

Etoposide

5-FU

10

4 Gy - Gamma ray

Camptothecin

H2O2

Bleomycin

2-DG

K2CrO4

5

NaAsO2 Paclitaxel Docetaxel

Trichostatin A

Heat Shock (47 C)

Vinblastin Colchicine Methotrexate Rotigotine Thapsigargin

0

Tunicamycin Antimycin A 4% EtOH

-5

2% EtOH

Oxamflatin

Apicidin

HC Toxin

-10

CdCl2

PC 2

## -10 -5 0 5 10

Hydroxyurea Ara C

# X

MMS

Cisplatin

Methotrexate

Thapsigargin

CdCl2

Vinblastine

Colchicine

Stauroporine

Paclitaxel Trichostatin A

OxamflatinApicidin

Heat Shock (47oC)

Docetaxel

HC Toxin

4% EtOH

2% EtOH

Antimycin A

Tunicamycin

MMS

Cisplatin

Camptothecin 5-FU

Hydroxyurea

Ara C

Etoposide

H2O2

NaAsO2

4 Gy - Gamma ray

K2CrO4

Bleomycin

2-DG

Etoposide

5-FU

10

4 Gy - Gamma ray

Camptothecin

H2O2

Bleomycin

2-DG

K2CrO4

5

NaAsO2 Paclitaxel Docetaxel

Trichostatin A Heat Shock (47 C) Vinblastin Colchicine Methotrexate Thapsigargin Staurosporine

0

CdCl2 Oxamflatin Apicidin

-5

HC Toxin

Tunicamycin

Antimycin A

4% EtOH

-10

2% EtOH

PC 2

## -10 -5 0 5 10

Hydroxyurea Ara C
